# Supplementary material for: Characteristic changes in EEG spectral powers of patients with opioid-use disorder as compared with those with methamphetamine- and alcohol-use disorders
Source: PLoS One. 2021 Sep 10;16(9):e0248794. doi: 10.1371/journal.pone.0248794 (PMC8432824; doi:10.1371/journal.pone.0248794)
Supplement: S5 File — (DOCX) [file pone.0248794.s005.docx]

Table 1: Breakdown of information of CTL group

| Subject ID | Age (year) | Sex | Ethnicity^1^ |
| --- | --- | --- | --- |
| C2 | 28 | M | W |
| C3 | 24 | F | W |
| C4 | 39 | M | H |
| C5 | 23 | F | W |
| C6 | 40 | M | H |
| C7 | 38 | F | W |
| C8 | 26 | M | W |
| C9 | 21 | F | W |
| C10 | 26 | M | H |
| C11 | 60 | M | W |
| C12 | 55 | M | W |
| C13 | 26 | M | W |
| C14 | 46 | M | W |
| C15 | 50 | M | W |
| C16 | 24 | M | W |
| C17 | 37 | F | W |
| C18 | 41 | F | W |
| C19 | 30 | M | W |
| C20 | 20 | M | W |
| C21 | 21 | F | W |

^1^W denotes White; H, Hispanic. No medications were reported at time of recording. No health issues were reported at the time of recording. The group consisted of 20 total cases; 13 males, 7 females. Mean age of 34 years.

Table 2: Breakdown of medical information of OUD patients

| Subject ID | Age (year) | Sex | Ethnicity^1^ | Drug^2^ | Years on drug | Other health issues | Medications at the time of testing |
| --- | --- | --- | --- | --- | --- | --- | --- |
| O2 | 33 | M | W | O, H | 14 | Bipolar disorder, unspecified | Buprenorphine |
| O3 | 29 | M | W | H | 6 | N/A | Buprenorphine; Gabapentin |
| O6 | 31 | F | W | H | 5 | N/A | Buprenorphine |
| O7 | 44 | M | W | H | 20 | Hepatitis C | None |
| O8 | 22 | F | W | H | 4 | Hepatitis C | Buprenorphine; Gabapentin; Quetiapine |
| O13 | 24 | M | Hi | H | 5 | Hepatitis C | None |
| O14 | 30 | M | W | O | 3 | N/A | Buprenorphine |
| O15 | 51 | M | W | O | 4 | Essential hypertension | Buprenorphine; Hydroxyzine |
| O17 | 56 | M | Hi | H | 5 | Mild intermittent asthma | Buprenorphine |
| O18 | 49 | M | W | O | 3 | Essential hypertension | Buprenorphine; Metoprolol |
| O26 | 35 | M | W | O, H | 15 | Psoriasis vulgaris | Buprenorphine |
| O27 | 45 | F | W | O | 10 | Essential hypertension; Gastroesophageal reflux disease | Buprenorphine; Pantoprazole |
| O28 | 45 | F | W | H | 3 | N/A | Buprenorphine; Gabapentin |
| O29 | 20 | M | W | H | 2 | N/A | None |
| O30 | 40 | M | W | O | 12 | N/A | Buprenorphine; Pantoprazole |
| O31 | 29 | M | W | H | 7 | N/A | Buprenorphine; Gabapentin; Hydroxyzine |
| O33 | 25 | F | W | H | 4 | N/A | Buprenorphine; Gabapentin; Hydroxyzine |
| O34 | 49 | M | Hi | O | 13 | Gastro-esophageal reflux disease; Essential hypertension; Testicular hypofunction | Buprenorphine; Gabapentin; Hydroxyzine |
| O35 | 22 | F | W | H | 4 | N/A | Buprenorphine |
| O26 | 19 | F | W | H | 4 | N/A | Buprenorphine |

^1^W denotes White; Hi, Hispanic.^2^M denotes morphine; H, heroin; O, oxycodone. The group consisted of 20 total cases; 13 males; 7 females. Mean age of 37 years.

Table 3: Breakdown of medical information of MUD patients

| Subject ID | Age (year) | Sex | Ethnicity^1^ | Years on drug | Other health issues | Medications at the time of testing |
| --- | --- | --- | --- | --- | --- | --- |
| A2 | 39 | M | W | 5 | N/A | Diazepam |
| A3 | 23 | M | H | 4 | N/A | None |
| A6 | 38 | M | B | 7 | Acute hepatitis C, without hepatic coma | Trazodone |
| A7 | 48 | M | W | 5 | Essential hypertension | Hydroxyzine |
| A9 | 27 | M | B | 2 | Gastro-esophageal reflux disease | Trazodone; Pantoprazole |
| A10 | 34 | M | W | 2 | Gastro-esophageal reflux disease | Diazepam |
| A11 | 32 | M | W | 10 | N/A | None |
| A13 | 25 | F | H | 2 | Mild intermittent asthma | Albuterol |
| A14 | 32 | M | W | 5 | Essential hypertension | Clonidine |
| A16 | 31 | F | W | 10 | N/A | Trazodone |
| A17 | 30 | F | W | 10 | N/A | None |
| A18 | 24 | F | W | 1 | N/A | Hydroxyzine |
| A22 | 20 | M | W | 3 | Myalgia | Hydroxyzine |
| A23 | 20 | M | W | 3 | N/A | Trazodone |
| A24 | 20 | M | W | 3 | N/A | Hydroxyzine; Mirtazapine |

^1^W denotes White; H, Hispanic; B, Black. The group consisted of 15 total cases; 11 males, 4 females. Mean age of 30 years.

Table 4: Breakdown of medical information of AUD patients

| Subject ID | Age (year) | Sex | Ethnicity^1^ | Years on drug | Other health issues | Medications at the time of testing |
| --- | --- | --- | --- | --- | --- | --- |
| A2 | 31 | M | W | 10 | Essential hypertension | Gabapentin |
| A3 | 57 | M | W | 20 | Essential hypertension; hyperlipidemia | Methocarbamol; Gabapentin; Losartan |
| A4 | 25 | F | W | 3 | N/A | Olanzapine; Pantoprazole |
| A5 | 31 | M | W | 12 | Gastro-esophageal reflux disease | Buspirone; Gabapentin; Pantoprazole |
| A8 | 49 | F | W | 20 | N/A | Hydroxyzine; Diazepam; Gabapentin |
| A9 | 41 | M | W | 25 | Essential hypertension | Diazepam; Gabapentin |
| A11 | 36 | F | W | 5 | N/A | Diazepam; Gabapentin |
| A13 | 30 | M | H | 2 | Mixed hyperlipidemia | Diazepam; Hydroxyzine |
| A15 | 23 | M | W | 3 | N/A | Gabapentin |
| A16 | 32 | M | W | 8 | N/A | Diazepam; Gabapentin |
| A18 | 37 | F | W | 3 | Gastro-esophageal reflux disease | None |
| A19 | 54 | M | W | 25 | Anemia | Diazepam; Carbamazepine |
| A20 | 23 | M | W | 5 | N/A | None |
| A22 | 44 | F | W | 10 | Essential hypertension | Diazepam; Gabapentin |
| A23 | 40 | F | H | 5 | Essential hypertension | Lisinopril; Carvedilol; Pantoprazole |
| A24 | 28 | M | W | 5 | N/A | Methocarbamol |
| A25 | 47 | M | W | 10 | Type II diabetes; essential hypertension; myalgia | Atorvastatin; Pioglitazone; Canagliflozin |
| A26 | 55 | M | W | 10 | Gastro-esophageal reflux disease | Levetiracetam; Diazepam |
| A27 | 46 | M | W | 3 | Mild intermittent asthma; essential hypertension | Diazepam; Gabapentin |
| A28 | 42 | M | W | 10 | N/A | None |
| A29 | 35 | M | W | 3 | N/A | None |
| A30 | 32 | M | H | 5 | Essential hypertension; asthma | Diazepam; Levetiracetam |
| A31 | 26 | M | W | 2 | N/A | Gabapentin |

^1^W denotes White; H, Hispanic; B, Black. The group consisted of 23 total cases; 17 males, 6 females. Mean age of 38 years.
