# Supplementary figures and images for: Characteristic changes in EEG spectral powers of patients with opioid-use disorder as compared with those with methamphetamine- and alcohol-use disorders
Source: PLoS One. 2021 Sep 10;16(9):e0248794. doi: 10.1371/journal.pone.0248794 (PMC8432824; doi:10.1371/journal.pone.0248794)

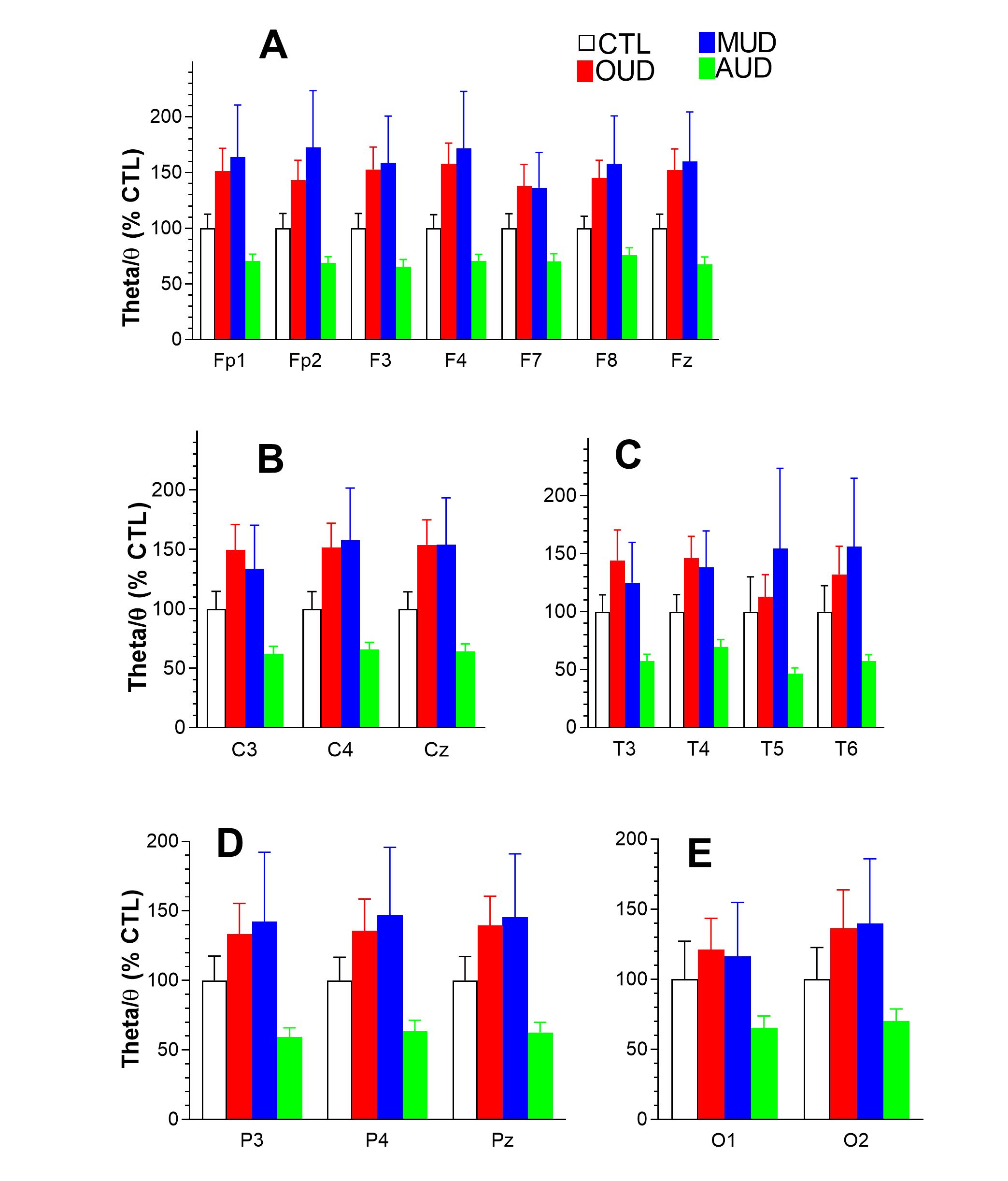

Supplement: S6 File — Data were expressed as % CTL A, Frontal. B, Central. C, Temporal. D, Parietal. E, Occipital. Overall, MUD or OUD theta/θ powers >CTL >AUD. However, OUD, MUD or AUD was not different from the CTL (P>0.05). (TIF) [file pone.0248794.s006.tif]

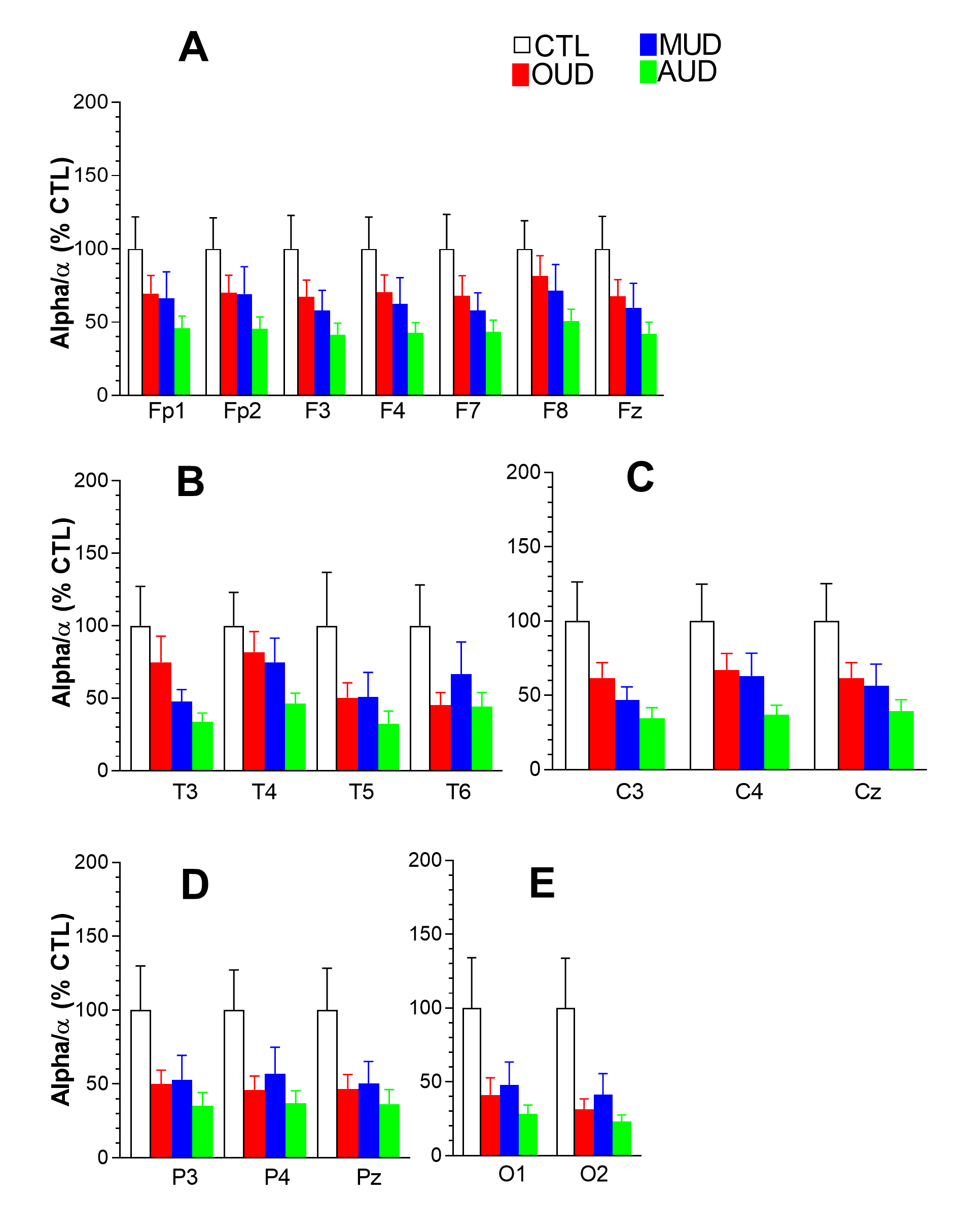

Supplement: S7 File — Data were expressed as % CTL. A, Frontal. B, Central. C, Temporal. D, Parietal. E, Occipital. Overall, CTL alpha/α power >OUD >MUD >AUD. OUD, MUD or AUD was not different from the CTL (P>0.05). (TIF) [file pone.0248794.s007.tif]

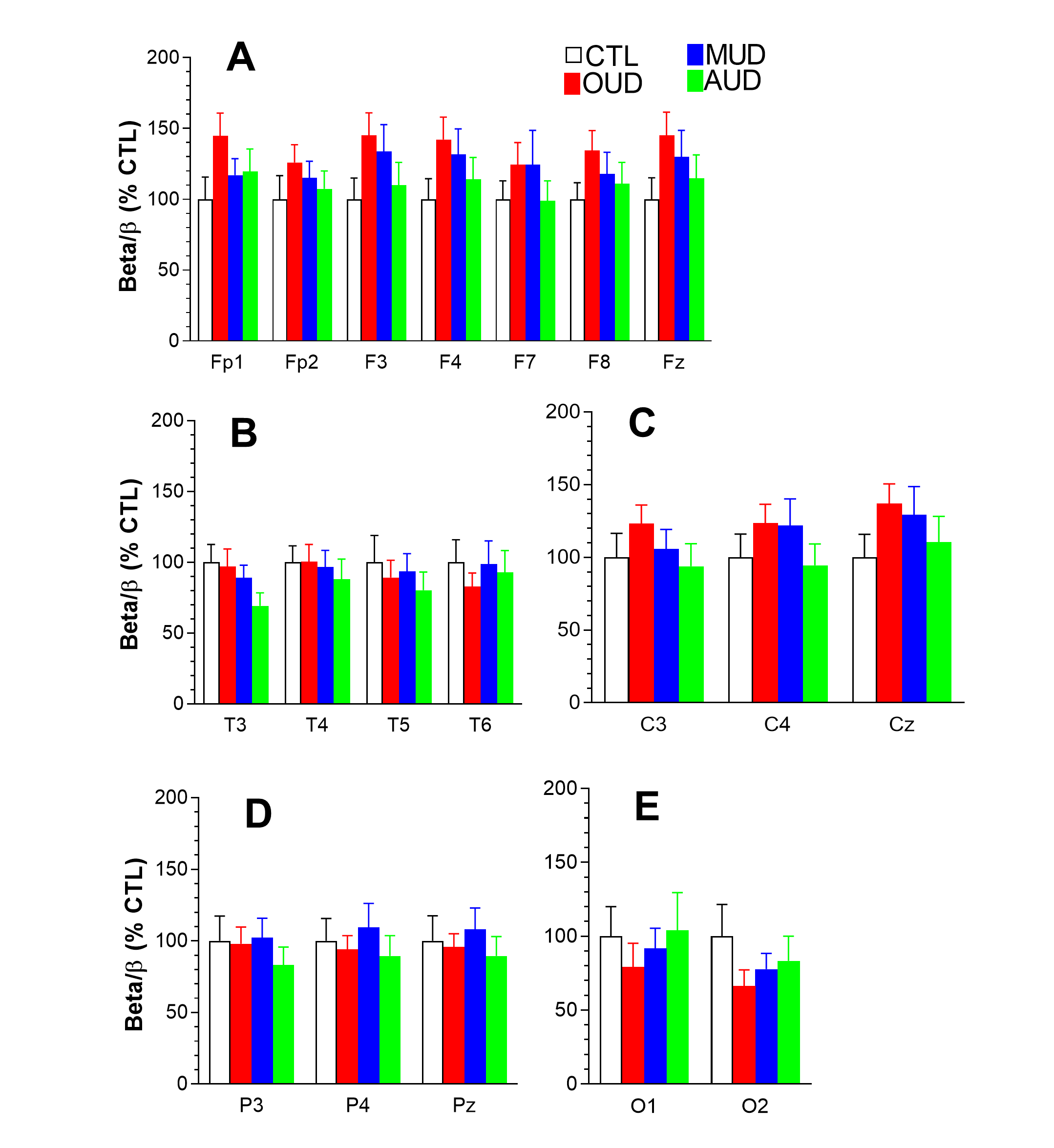

Supplement: S8 File — Data were expressed as % CTL. A, Frontal. B, Central. C, Temporal. D, Parietal. E, Occipital. OUD, MUD or AUD was not different from the CTL (P>0.05). (TIF) [file pone.0248794.s008.tif]

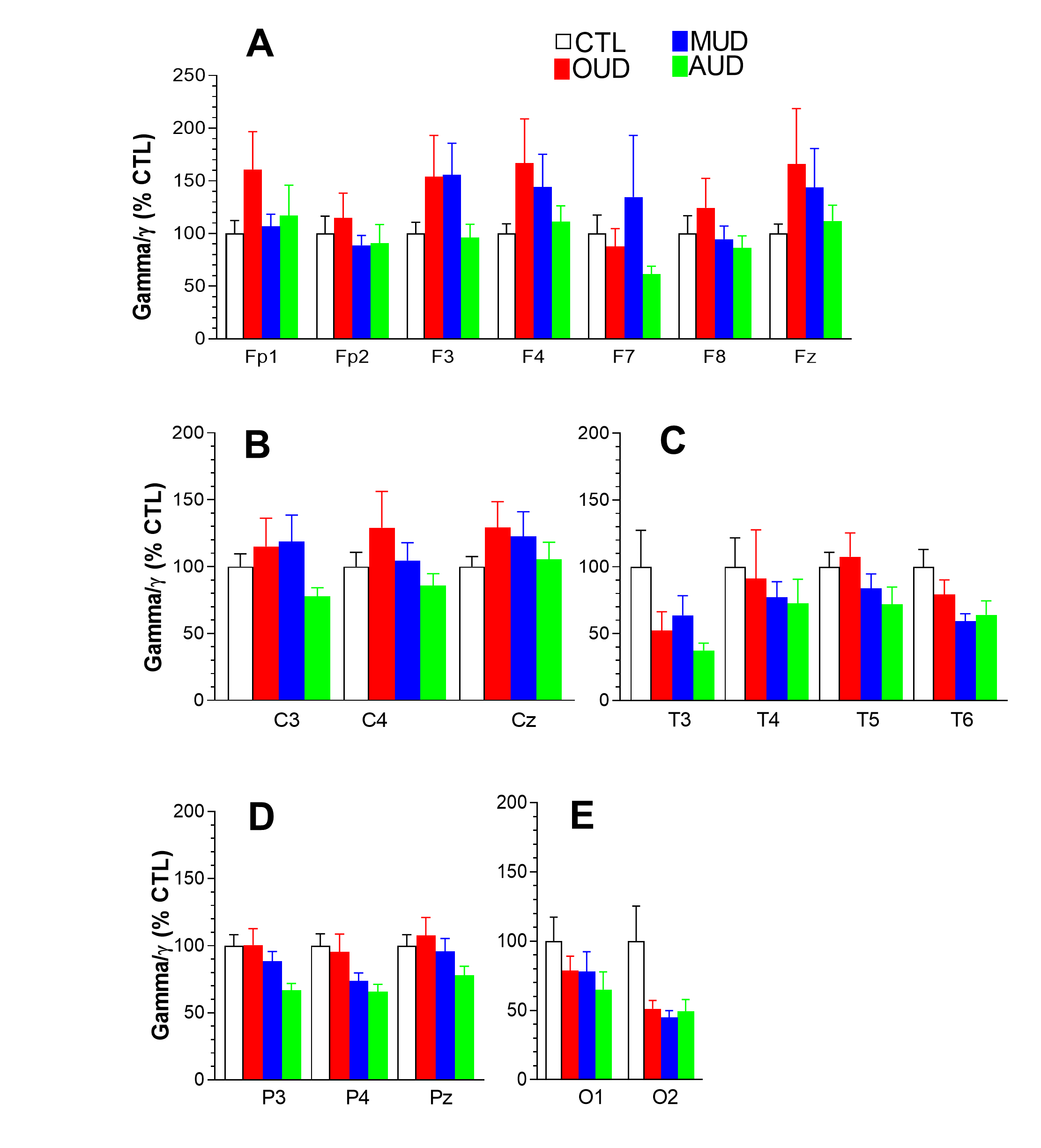

Supplement: S9 File — Data were expressed as % CTL. A, Frontal gamma/γ powers. B, Central gamma/γ powers. C, Temporal gamma/γ powers. D, Parietal gamma/γ powers. E, Occipital gamma/γ powers. OUD, MUD or AUD was not different from the CTL (P>0.05). (TIF) [file pone.0248794.s009.tif]
