# Supplementary material for: Characteristic changes in EEG spectral powers of patients with opioid-use disorder as compared with those with methamphetamine- and alcohol-use disorders
Source: PLoS One. 2021 Sep 10;16(9):e0248794. doi: 10.1371/journal.pone.0248794 (PMC8432824; doi:10.1371/journal.pone.0248794)
Supplement: Attachment — Submitted filename: Response to Reviewers.docx [file pone.0248794.s010.docx]

Dear Dr. Mumtaz,

We would like to thank you, Dr. Attarian, and reviewer #2 for your time and efforts in reviewing our manuscript entitled “Characteristic changes in EEG spectral powers of patients with opioid-use disorder as compared with those with methamphetamine- and alcohol-use disorders”.

Questions and concerns have been answered in a point-to-point manner. We also conducted the statistical analysis with smaller case numbers as suggested, however, finding that results became weaker. Therefore, we kept the data as was.

The authors received no specific funding for this work. Please change the online submission form on our behalf. We have resubmitted the revised manuscript and hope the revision would be satisfactory for consideration for publishing.

Sincerely,

Rui Tao, Ph. D

=====================================================

Response to editorial questions by Dr. Mumtaz

***Q1.*** *Please ensure that your manuscript meets PLOS ONE's style requirements*

**Reply:** The style has been followed as instructed.

***Q2****. In the Methods section and the online submission form, please provide additional information about the patient records used in your retrospective study.*

**Reply:** Information was specified in the “Method” section, pg5 line 119-123

***Q3****. ……stating the following financial disclosure*

**Reply**: Checked

***Q4****. Your ethics statement should only appear in the Methods section of your manuscript*

Reply: checked.

====================================================

Response to Dr. Hrayr Attarian

**Q5**. ……. *the results would be even more relevant and valuable if there was an analysis done of the EEG spectra in wakefulness vs REM sleep vs NREM sleep. At least there should be a comparison between wakefulness and sleep as alcohol, opiates and stimulants can affect neurophysiological function in sleep differently than they do in wakefulness*

**Reply**: We totally agree with the importance to show the difference between wakefulness and sleep in patients with opioid use disorder in contrast to those with alcohol or stimulant use disorders. However, there is no sleep data available for us. Therefore, we could not conduct the comparative study. The comments are very valuable for future direction.

The retrospective EEG data used in the present studies refers only at the eye-closed (wakeful) state.

====================================================

Response to Reviewer #2: 

**Q6**. *Data availability: Data location has not been listed. As such, data is not available.*
**Reply**: Thanks for the comment. In this revision, every raw data was attached to supplementary sections S1-S5.

**Q7**. ……. *Were the data collected only once? Did the subjects return for multiple recordings?*

**Reply**: Only one time of data prior to rehab treatment was approved to be used in the present study. Patients did return after rehab treatment. However, data after rehab treatment were not approved for disclosure.

**Q8.** *Were their tasks performed?*

**Reply**: No tasks performed. The eye closed, and subject seated calmly without any activity or movement.

**Q9***……Were the powerline noise removed from the data?*

**Reply**: Yes, it was removed. Additionally, artifacts (non-EEG) were also removed from the data, as described on pg6 line 149-151 “…….. raw data was edited using the editing tool within the NeuroGuide software to remove physical artifacts (including eye movement, jaw movement, and gross movement)……”

**Q10**. *Out of 350 Opioid, 450 Alcohol and 100 Meth, selection of only about 20 subjects per group seems to be limited by the control group. This is the main weakness of the paper*.

**Reply**: We appreciate the reviewer’s concern. However, most patients in our database were found to be multi-drug users. Therefore, most of them should be excluded from the study. Patients with neurodegenerative diseases (e.g., PD and MD) should also be excluded. Only a few dozen patients met the criteria for meaningful retrospective data analysis.

Our primary goal in this study was to characterize EEG in patients with opioid use disorder (OUD). For this reason, 20 OUD patients were compared with sex- and age-matched healthy control. We believe that readers might feel more interested in knowing the difference in EEG between OUD and stimulants (MUD) or alcohol (AUD). Therefore, we compared OUD with MUD or AUD. Although we could not get sex- and age-matched (stimulant/MUD or alcohol/AUD) data, we think the results were still valuable, even though, we had only 15 MUD patients.

**Q11**. *The authors can divide the A, M and O groups to smaller groups of 20 subjects and see if an average of all subgroups match with the current conclusions of the paper. This may increase statistical significance of the results.*

**Reply**: We conducted a statistical analysis with a smaller number of cases as suggested to see if statistical significance of the results would increase or not. Because we had only 15 MUD cases, we had to remove 5 cases out of the CTL (C4, C7, C14, C12, and C15), 5 from the opioids (O7, O15, O17, O18, and O34), and 7 out of the alcohol (A3, A8, A19, A25, A27, and A28) to have 15 cases per every group. All groups were analyzed. Please see the table below.

|  | CTL (N=15) | OUD (N=15) | MUD (N=15) | AUD (N=15) |  |
| --- | --- | --- | --- | --- | --- |
| Age (years) | 29 (±8) | 30 (±9) | 29 (±8) | 33 (±6) |  |
| Sex (M/F) | 8/7 | 8/7 | 11/4 | 10/5 |  |
| Duration of substances used (years) | 0 | 6 (±4) | 5 (±3) | 6 (±7) |  |

We started to re-examine delta/δ powers presented in fig 3B. The ANOVA was used to analyze 4 groups of absolute delta/δ values of the F3 electrode, revealing significant difference between 4 groups, showing F(3,56) =3.203, P =0.03. We also analyzed the percentage values and had the same results. We next conducted *post-hoc* test analysis revealing that changes in opioids/OUD, stimulants/MUD or alcohol/OUD were not different from CTL (see figure below). The statistical significance of the results decreased. Given that increases in numbers of cases would improve the significance of statistical analysis, we decided to keep the original analysis (i.e., 20 cases in CTL; 20/opioid, 15/stimulants, and 23/alcohol) showing F(3,74) =6.07, P). =0.0009.

To clarify the question for readers, we added three new sentences on pg11 line251-254, stating that ‘The normalized data appear to have the same response pattern as……absolute value’

In addition to F3, all 19 electrodes were re-examined using smaller numbers as shown below. Similarly, figures C’-G’ displayed the same tendency as figure 3C-G in the manuscript although case numbers reduced. However, no electrode was found to have statistical significance. For this reason, we kept the case numbers in the manuscript as was.

**Q12**. *While absolute band powers have been analyzed, it may be useful to analyze relative band powers. These have played a role in discriminating two classes of EEG signals such as pre-ictal and inter-ictal in epilepsy. Relative band powers can be calculated by dividing every subjects band power by the total power spectral density. See for example,
Z. Zhang et al, "Low-Complexity Seizure Prediction From iEEG/sEEG using Spectral Power and Ratios of Spectral Power," 2016.*

**Reply**: This is also a good point raised by the reviewer. Analysis of absolute values could be simple but potentially create data bias due to individual differences, which was correctly emphasized by Zhang et al 2016. To do this, EEG changes in seizure attack could be normalized to the pre-seizure EEG powers, which is a perfect approach for the seizure analysis.

Regarding our patients with substance use disorders (SUD), pre-SUD data were not available in the database. Therefore, we could not calculate changes relative to pre-SUD levels when they were still mentally healthy. Fortunately, there were 20 healthy CTL included in the study. Those CTL data could be used as pre-SUD data. Specifically, in our manuscript, relative changes (% CTL) were determined with CTL EEG powers, displayed in fig 3A-G, and also supplementary S2-5. In other words, our data analysis in the manuscript considered both absolute and relative values. We found that statistical analyses over relative changes were the same as the absolute values, indicating no difference. Absolute values were used in figures 4-8 for a direct comparison of our results with other labs (see details on pg11 line251-254)

Alternatively, relative powers of individual/group electrodes can be divided by the total powers of all 19 electrodes. Please note that the total powers presented in fig 8, showed significant difference in EEG between users. Although we haven’t divided individual/group electrodes by the total powers, we will include this idea in future analysis.

***Q13****. While the statistical behavior is useful and should be published, the community has focused on binary classification. This could be considered as future work. As one example of such classification, the authors may refer to:
B. Sen et al., "Sub-Graph Entropy based Network Approaches for Classifying Adolescent Obsessive-Compulsive Disorder from Resting-State Functional MRI," 2020.*

**Reply**: We appreciate this suggestion.
