# Supplementary material for: Characteristic changes in EEG spectral powers of patients with opioid-use disorder as compared with those with methamphetamine- and alcohol-use disorders
Source: PLoS One. 2021 Sep 10;16(9):e0248794. doi: 10.1371/journal.pone.0248794 (PMC8432824; doi:10.1371/journal.pone.0248794)
Supplement: Attachment — Submitted filename: Reponse to Reviewers.docx [file pone.0248794.s011.docx]

Dear Dr. Mumtaz,

We would like to thank you, Dr. Attarian, and reviewer #2 for your time and efforts in reviewing our manuscript entitled “Characteristic changes in EEG spectral powers of patients with opioid-use disorder as compared with those with methamphetamine- and alcohol-use disorders”.

The references cited in the manuscript has been reviewed. The response to reviewer#2 has been integrated into the context of this manuscript as suggested.

We have resubmitted the revised manuscript and hope the revision would be satisfactory for consideration for publishing.

Sincerely,

Rui Tao, Ph. D

=====================================================

Response to editorial requirement

Q1. *Please review your reference list to ensure that it is complete and correct. If you have cited papers that have been retracted, please include the rationale for doing so in the manuscript text, or remove these references and replace them with relevant current references. Any changes to the reference list should be mentioned in the rebuttal letter that accompanies your revised manuscript. If you need to cite a retracted article, indicate the article’s retracted status in the References list and also include a citation and full reference for the retraction notice.*

Reply: The references cited in the manuscript has been reviewed in a one to one manner. Specifically, the references were copy-pasted to the NIH PUBMED website. There was no information indicating retraction of references cited in the manuscript.

Response to reviewer #2

Q2. …*the response should be included in main part of the paper. Future study direction with respect features and classifiers should be pointed out*

**Reply:** the response has been included into the context of the manuscript. Details are as follows.

| **Questions and concerns** | **Pages, lines and response located in the manuscript** |
| --- | --- |
| *Were the data collected only once? Did the subjects return for multiple recordings?* | on pg6 lines 150-151  “Only one time of data prior to rehab treatment was approved by the IRAs to be used in the present study” |
| *“Were their tasks performed?”* | on pg6 lines 135-136  “Eyes closed, and subject seated calmly without any activity or movement” |
| **“***Were the powerline noise removed from the data?* | on pg6 line 152-154  “…….. raw data was edited using the editing tool within the NeuroGuide software to remove physical artifacts (including eye movement, jaw movement, and gross movement)… |
| *The authors can divide the A, M and O groups to smaller groups of 20 subjects and see if an average of all subgroups match with the current conclusions of the paper. This may increase statistical significance of the results* | *On pg15-16*  ……(see details in fig 3, and also supplementary S1-S4). One explanation for such failure could be that the retrospective analysis was not designed as a one-to-one matched case control study. It was likely to be that the statistical power would be increased if the subgroups were matched. To test such possibility, subjects were reduced to 15 cases/subgroup for the age- and sex-matched design. Delta/δ powers presented in fig 3B were re-examined with the ANOVA, revealing significant difference between 4 groups, F_(3,56)_ =3.203, P =0.03. However, the *post-hoc* analysis revealed that changes in OUD, MUD or AUD were not different from CTL, suggesting the one-to-one matched design could not fully explain the failure at statistical analysis.  Alternatively, a single electrode covered only small brain areas in terms of EEG spatial sizes and thus relatively small numbers of neurons affected…. |
| *While absolute band powers have been analyzed, it may be useful to analyze relative band powers. These have played a role in discriminating two classes of EEG signals such as pre-ictal and inter-ictal in epilepsy. Relative band powers can be calculated by dividing every subjects band power by the total power spectral density. See for example, Z. Zhang et al, "Low-Complexity Seizure Prediction From iEEG/sEEG using Spectral Power and Ratios of Spectral Power," 2016.* | *On pg 10 line247-pg11 line 253*  Next, the EEG waves were transformed into amplitude powers expressed as µV^2^, as shown in the left panel of Fig 3B. The difference in delta/δ amplitude powers was statistically significant [F_(3, 74)_ =6.07, P =0.0009]. However, *post-hoc* analysis indicates that only OUD, but not MUD or AUD, reached statistical significance difference from the CTL. Analysis of absolute values could be simple but potentially create data bias due to individual differences. To minimize such possibility, data were normalized into %CTL. |
| *While the statistical behavior is useful and should be published, the community has focused on binary classification. This could be considered as future work. As one example of such classification, the authors may refer to: B. Sen et al., "Sub-Graph Entropy based Network Approaches for Classifying Adolescent Obsessive-Compulsive Disorder from Resting-State Functional MRI," 2020.* | *On pg19, line 496-498*  ….Despite such importance, it cannot exclude the possibility of bias, so other biomarkers and binary classification are needed to be included in the future study by which the conclusion can be alternatively corroborated… |
